# *Xanthomonas hortorum* pv. *gardneri* TAL effector AvrHah1 is necessary and sufficient for increased persistence of *Salmonella enterica* on tomato leaves

**DOI:** 10.1038/s41598-022-11456-6

**Published:** 2022-05-04

**Authors:** Kimberly N. Cowles, Anna K. Block, Jeri D. Barak

**Affiliations:** 1 Department of Plant Pathology, University of Wisconsin-Madison, Madison, WI USA; 2 Center for Medical, Agricultural, and Veterinary Entomology, U.S. Department of Agriculture-Agricultural Research Service, Gainesville, FL USA

**Keywords:** Plant molecular biology, Bacterial host response, Bacteriology

## Abstract

*Salmonella enterica* is ubiquitous in the plant environment, persisting in the face of UV stress, plant defense responses, desiccation, and nutrient limitation. These fluctuating conditions of the leaf surface result in *S. enterica* population decline. Biomultipliers, such as the phytopathogenic bacterium *Xanthomonas hortorum* pv. *gardneri (Xhg)*, alter the phyllosphere to the benefit of *S. enterica*. Specific *Xhg-*dependent changes to this niche that promote *S. enterica* persistence remain unclear, and this work focuses on identifying factors that lead to increased *S. enterica* survival on leaves. Here, we show that the *Xhg* transcription activator-like effector AvrHah1 is both necessary and sufficient for increased survival of *S. enterica* on tomato leaves. An *Xhg avrHah1* mutant fails to influence *S. enterica* survival while addition of *avrHah1* to *X. vesicatoria* provides a gain of function. Our results indicate that although *Xhg* stimulates a robust immune response from the plant, AvrHah1 is not required for these effects. In addition, we demonstrate that cellular leakage that occurs during disease is independent of AvrHah1. Investigation of the interaction between *S. enterica, Xhg,* and the plant host provides information regarding how an inhospitable environment changes during infection and can be transformed into a habitable niche.

## Introduction

*Salmonella enterica* is a human enteric pathogen that causes disease in approximately 1.2 million Americans annually (CDC). Numerous multi-state outbreaks of salmonellosis have occurred from the consumption of fresh produce every year since 2000 (National Outbreak Reporting System (NORS;^1–4^)). *S. enterica* survives on multiple agricultural crops after root or leaf immigration^5–7^, and the bacteria persist in soil for months^6,8,9^. Despite the continued isolation of *S. enterica* from the agricultural environments associated with outbreaks, *S. enterica* has relatively poor fitness in the plant environment. Bacterial populations steadily decline in the phyllosphere, or above-ground parts of plants^8,9^.

The rapidly fluctuating conditions on the leaf surface provide a harsh environment for epiphytic bacteria, and survival depends on the ability to adapt to abrupt changes in UV irradiation and the availability of water and nutrients. Upon arrival to the leaf surface, *S. enterica* migrates to protected sites like trichomes, stomates, hydathodes, and epidermal cell wall junctions^7,10–12^. These locations are often associated with a local increase in nutrient abundance due to cracks or leakage through the cuticular layer and may provide protection from desiccation or UV stress^10,13^. The accessibility of these sites likely explains both how *S. enterica* persists on leaves over long periods of time and why eventually populations decline. This failure to replicate faster than cellular death is most likely because *S. enterica* cannot access the nutrient-rich interior of plant tissues on its own.

Recent studies involving *S. enterica* colonization of plants have centered on the identification and characterization of biomultipliers, factors that lead to increased *S. enterica* survival. *S. enterica* exploits changes to the plant environment imparted by other organisms, including infestation with phytophagous insects and infection with bacterial phytopathogens^14–22^. Plants infected with *Xanthomonas* spp. lead to increased persistence of *S. enterica* on tomato (*Solanum lycopersicum*) leaves^17,18^. Four lineages of *Xanthomonas* cause bacterial spot of tomato: *X. hortorum* pv. *gardneri* (hereafter referred to as *Xhg*)*, X. euvesicatoria* pv. *euvesicatoria, X. euvesicatoria* pv. *perforans,* and *X. vesicatoria*^23–25^. These xanthomonads colonize tomato leaf surfaces as epiphytes and enter plant tissue through natural openings to circumvent the epidermis, the first layer of plant defense. Once inside, *Xanthomonas* colonizes the apoplast where it multiplies and eventually spreads to neighboring leaves, and ultimately other plants. Bacterial spot disease manifests as lesions on leaves, fruit, and stems of tomato plants and ultimately leads to loss of leaves and damaged fruit, resulting in substantial agricultural impact^23,24^.

Although four *Xanthomonas* lineages cause the same disease on tomato, only a subset of these lineages has a beneficial effect on *S. enterica* populations. In plants colonized by *Xhg, X. euvesicatoria* pv. *euvesicatoria,* or, to a lesser extent, *X. euvesicatoria* pv. *perforans*, *S. enterica* populations on tomato leaves remain steady or even increase over time^17^. Contrastingly, *S. enterica* populations decline in the presence of *X. vesicatoria* or on healthy plants^17^. Core Type III secretion system (T3SS) effectors are shared among the four *Xanthomonas* species, yet each species also has unique effectors. Of particular interest for this study, *Xhg* has a transcription activator-like effector (TALE) AvrHah1 that is not found in *X. vesicatoria*^26^. TALEs induce host transcription by directly binding to the promoter region of target genes using a modular, central repeat domain that recognizes specific host effector binding elements ^27,28^. *Xhg* AvrHah1 has over 4,000 potential, predicted binding sites in tomato^29^.

AvrHah1 induces a water soaking phenotype during disease^29,30^, which is characterized by dark green lesions on leaves that reflect an influx of fluid into the apoplast. This water soaking is not observed in *X. vesicatoria-*infected tissues^26^. In tomato, AvrHah1 is required for the direct induction of two beta helix loop helix (bHLH) transcription factors, bHLH3 and bHLH6^29^. The spike in transcription for these two regulators leads to indirect induction of at least two additional downstream genes, including a pectate lyase (PL) and pectinesterase (PE)^29^. The current model suggests that PL alters hygroscopicity in the plant cell wall by releasing oligosaccharides, resulting in an influx of fluid through breaks in the epidermis and the observed water-soaked phenotype^29^. Because the *Xhg avrHah1* mutant reaches the same population level as wildtype *Xhg in planta*, it is thought that AvrHah1 is involved in pathogen dispersal or entry into leaf tissue as opposed to acquisition of nutrients during infection^29^.

In this study, we tested the hypothesis that AvrHah1 defines the differential ability between *Xhg* and *X. vesicatoria* to enable *S. enterica* persistence in the phyllosphere. We showed that lack of this effector reduced the ability of *Xhg* to promote *S. enterica* persistence on tomato leaves*.* Correspondingly, the addition of *avrHah1* to *X. vesicatoria* allowed this phytopathogen to promote *S. enterica* survival. The data presented here provide evidence that AvrHah1-dependent water soaking may be the mechanism by which the phytopathogenic bacterium benefits the human enteric pathogen.

## Results

### AvrHah1 is both necessary and sufficient for *Xhg*-dependent effects on *S. enterica* persistence

**Figure 1:**
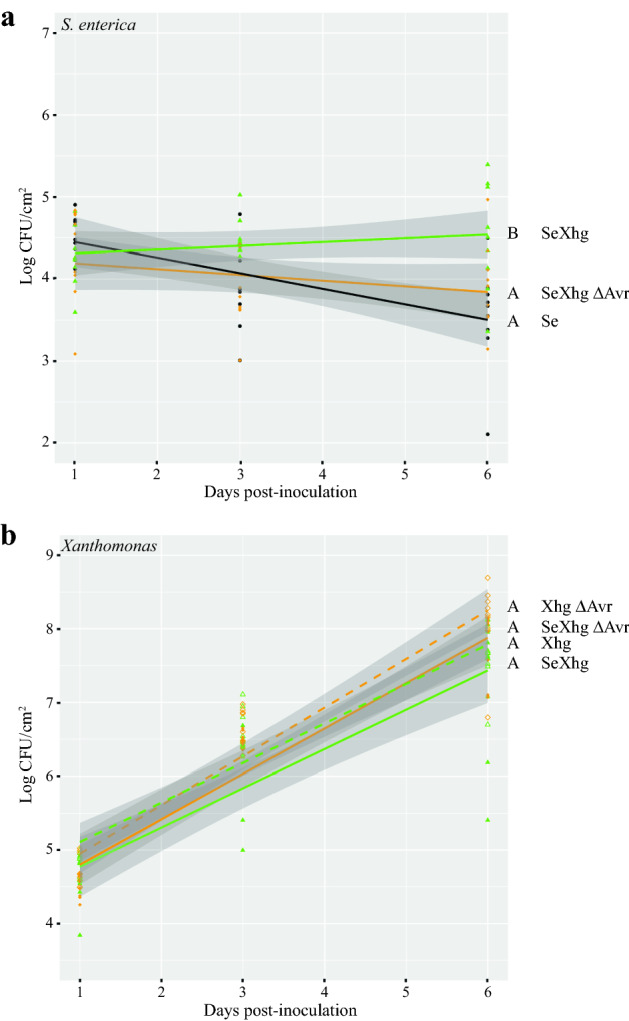
TALE AvrHah1 is required for *Xhg*-dependent effects on *S. enterica* persistence. Bacterial populations (*S. enterica*, (**a**); *Xanthomonas* (**b**)) on tomato leaves were monitored following treatment with *S. enterica* (Se; black circles), *Xhg* (Xhg; open green triangles), *Xhg avrHah1*^ΔDBD^ mutant (Xhg ΔAvr; open orange diamonds), *S. enterica* + *Xhg* (SeXhg; closed green triangles), or *S. enterica* + *Xhg avrHah1*^ΔDBD^ mutant (SeXhg ΔAvr; closed orange diamonds). All data points from three independent experiments are presented as log CFU/cm^2^. Lines (black, *S. enterica,* Se; dashed green, *Xhg,* Xhg; dashed orange, *Xhg avrHah1*^ΔDBD^ mutant, Xhg ΔAvr; solid green, *S. enterica* + *Xhg,* SeXhg; solid orange, *S. enterica* + *Xhg avrHah1*^ΔDBD^ mutant, SeXhg ΔAvr) correspond to a linear regression model with 95% confidence intervals represented by the shaded areas. Letters denote significant differences between treatments for the regression lines according to the linear regression model (*P* < 0.01). Combining three independent experiments, n = 9 plants per treatment per time point.

Previously, we had shown that infection with *Xhg* promotes survival of *S. enterica* on the leaves of tomato plants^19^. To determine the importance of AvrHah1 for *Xhg*-dependent effects on *S. enterica*, we examined the effects of co-inoculation with the *Xhg* AvrHah1 DNA-binding domain mutant *avrHah1*^ΔDBD^ ^30^. Bacterial populations were monitored on tomato plants dip-inoculated with *S. enterica* in the presence or absence of wildtype *Xhg* or the *Xhg avrHah1*^ΔDBD^ mutant. The presence of wildtype *Xhg* led to approximately ten-fold higher *S. enterica* populations than the *S. enterica* alone treatment by 6 dpi (Fig. 1a; P = 0.0058). Conversely, co-inoculation of *S. enterica* with the *Xhg avrHah1*^ΔDBD^ mutant had no significant impact on *S. enterica* (Fig. 1a; P = 0.889). *Xanthomonas* populations were monitored over time, and there were no significant differences between treatments (Fig. 1b; P > 0.556 for all comparisons).

**Figure 2:**
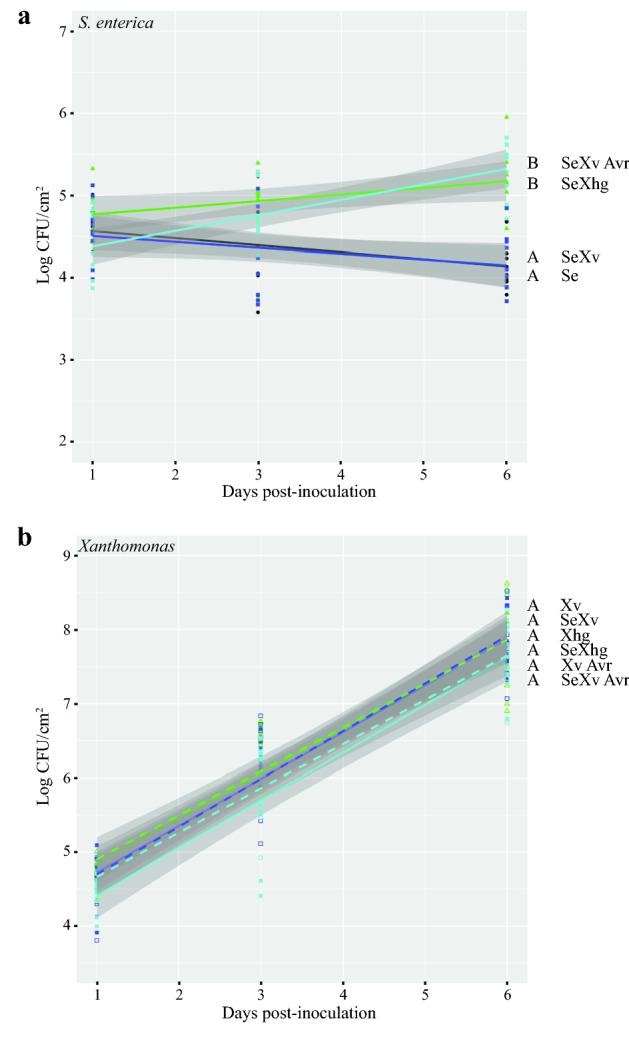
AvrHah1 is sufficient for *Xanthomonas*-dependent increases in *S. enterica* populations. Bacterial populations (*S. enterica*, (**a**); *Xanthomonas* (**b**)) on tomato leaves were monitored following treatment with *S. enterica* (Se; black circles), *Xhg* (Xhg; open green triangles), *X. vesicatoria* pUFR034 (Xv; open blue squares), *X. vesicatoria* pUFR034 (*avrHah1*) (Xv Avr; open cyan squares), *S. enterica* + *Xhg* (SeXhg; closed green triangles), *S. enterica* + *X. vesicatoria* pUFR034 (SeXv; closed blue squares), or *S. enterica* + *X. vesicatoria* pUFR034 (*avrHah1*) (SeXv Avr; closed cyan squares). All data points from three independent experiments are presented as log CFU/cm^2^. Lines (black, *S. enterica,* Se; dashed green, *Xhg,* Xhg; dashed blue, *X. vesicatoria* pUFR034, Xv; dashed cyan, *X. vesicatoria* pUFR034 (*avrHah1*), Xv Avr; solid green, *S. enterica* + *Xhg,* SeXhg; solid blue, *S. enterica* + *X. vesicatoria,* SeXv*;* solid cyan, *S. enterica* + *X. vesicatoria* pUFR034 (*avrHah1*), SeXv Avr) correspond to a linear regression model with 95% confidence intervals represented by the shaded areas. Letters denote significant differences between treatments for the regression lines according to the linear regression model (*P* < 0.01). Combining three independent experiments, n = 9 plants per treatment per time point.

To determine if *avrHah1* is sufficient for *Xanthomonas*-mediated effects on *S. enterica* persistence, *X. vesicatoria,* which does not impact *S. enterica* persistence, was transformed with a plasmid carrying the *avrHah1* gene (pUFR034 (*avrHah1*)) and tested for its effects on *S. enterica*. Bacterial populations were determined in tomato plants dip-inoculated with *S. enterica* in the presence or absence of *Xhg, X. vesicatoria* + pUFR034 (vector alone control), or *X. vesicatoria* + pUFR034 (*avrHah1*). Co-inoculation of *Xhg* or *X. vesicatoria* + pUFR034 (*avrHah1*) with *S. enterica* resulted in approximately ten-fold higher *S. enterica* populations at 6 dpi than in plants co-inoculated with *X. vesicatoria* + pURF034 and *S. enterica* or inoculated with *S. enterica* alone (Fig. 2a; *Xhg* P = 6.40 × 10^−7^ and 1.74 × 10^−6^, respectively; *X. vesicatoria* + pUFR034 (*avrHah1*) P = 8.17 × 10^−5^ and 0.00019, respectively). There was no significant difference between the *X. vesicatoria* + pURF034 and *S. enterica* alone treatments (P = 0.8203). *Xanthomonas* populations were monitored over time, and there were no significant differences between treatments (Fig. 2b; P > 0.97). These data demonstrate that the presence of AvrHah1 in *Xanthomonas* spp. can increase the persistence of *S. enterica* in co-inoculated leaves.

### The AvrHah1 targets ***bHLH3***, ***bHLH6***, ***PL***, and ***PE ***are transcribed to similar levels in plants infected with wildtype ***Xhg ***or the ***Xhg avrHah1***^ΔDBD^ mutant

Previous work has shown that AvrHah1 activates expression of multiple tomato genes, including two direct targets *bHLH3* and *bHLH6* and two indirect targets *PL* and *PE*^29^. These targets provide the foundation for the current model of AvrHah1 water soaking. To examine whether these targets play a role in the mechanism by which *Xhg* enhances *S. enterica* persistence, we monitored transcription of the genes in leaf samples after inoculation with *S. enterica* in the presence or absence of wildtype *Xhg* or the *Xhg avrHah1*^ΔDBD^ mutant. Under our experimental conditions, plants infected with the *Xhg avrHah1*^ΔDBD^ mutant had the same or higher levels of the four targets compared to plants infected with wildtype *Xhg* at 1, 3, and 6 dpi (Table 1). In contrast, published results show that wildtype *Xhg* Xg153 significantly induces transcription of these genes (~ 90–1,300-fold) compared to a corresponding *avrHah1*^ΔDBD^ mutant when infiltrated into tomato Heinz 1706 leaves for 48 hours^29^. To determine the effect of inoculation method (dip-inoculation vs infiltration) on our differing results, we infiltrated MoneyMaker tomato leaves with *Xhg* 444 wildtype and *avrHah1*^ΔDBD^ mutant following published protocols^29^. Leaf samples were collected 48 h post-infiltration, and plant gene expression was measured using quantitative PCR (qRTPCR). As with the dip-inoculation experiments, there were no significant reductions in plant gene expression in plants inoculated with the *Xhg avrHah1*^ΔDBD^ mutant compared to plants inoculated with wildtype *Xhg* for *bHLH3, PL,* or *PE* (Table 1). The only observed reduction in expression in plants inoculated with the *Xhg avrHah1*^ΔDBD^ mutant was a ~ 25-fold reduction in *bHLH6* transcription compared to plants inoculated with wildtype *Xhg* (Table 1). These data indicate that AvrHah1 may target other host genes in the mechanism that leads to increased *S. enterica* persistence in this system.

**Table 1:** Transcription of AvrHah1 targets in tomato leaves after infection with the *Xhg avrHah1*^ΔDBD^ mutant.

Gene	Dip-inoculation			Infiltration
	1 dpi^a^	3 dpi	6 dpi	48 h
*bHLH3*	3.28 ± 2.60	1.10 ± 0.21	1.21 ± 0.82	1.71 ± 0.14*^b^
*bHLH6*	1.88 ± 0.70*	0.76 ± 0.17	1.52 ± 1.26	0.04 ± 0.01*
*PL*	2.00 ± 1.42	1.20 ± 0.39	4.38 ± 4.75	9.99 ± 2.17*
*PE*	1.63 ± 1.02	0.85 ± 0.26	2.49 ± 2.71	0.90 ± 0.19

^a^Gene expression is displayed as the relative expression ratio of transcription levels after infection with the *Xhg avrHah1*^ΔDBD^ mutant using the plant response to wildtype *Xhg* as the calibrator (i.e., the response to wildtype *Xhg* is set to 1.0).

^b^Asterisks indicate statistical differences in plant gene expression after treatment with the *Xhg avrHah1* mutant compared to plant gene expression after treatment with wildtype *Xhg* using t-test (*P* < 0.05).

### *Xhg* and *X. vesicatoria* elicit different immune responses in tomato leaves, which are further affected by co-inoculation with *S. enterica*

To test the hypothesis that infection with *Xhg* alters the plant immune response to the benefit of *S. enterica,* plant defense gene expression was monitored over time in tomato leaves inoculated with *S. enterica*, *Xhg, X. vesicatoria,* or a combination of each xanthomonad with *S. enterica*. The SA-inducible pathogenesis related protein gene *pr1a1* and the JA-inducible proteinase inhibitor gene *pin1* were used as established markers^31,32^ to indirectly monitor these two plant defense pathways with qRTPCR.

**Figure 3:**
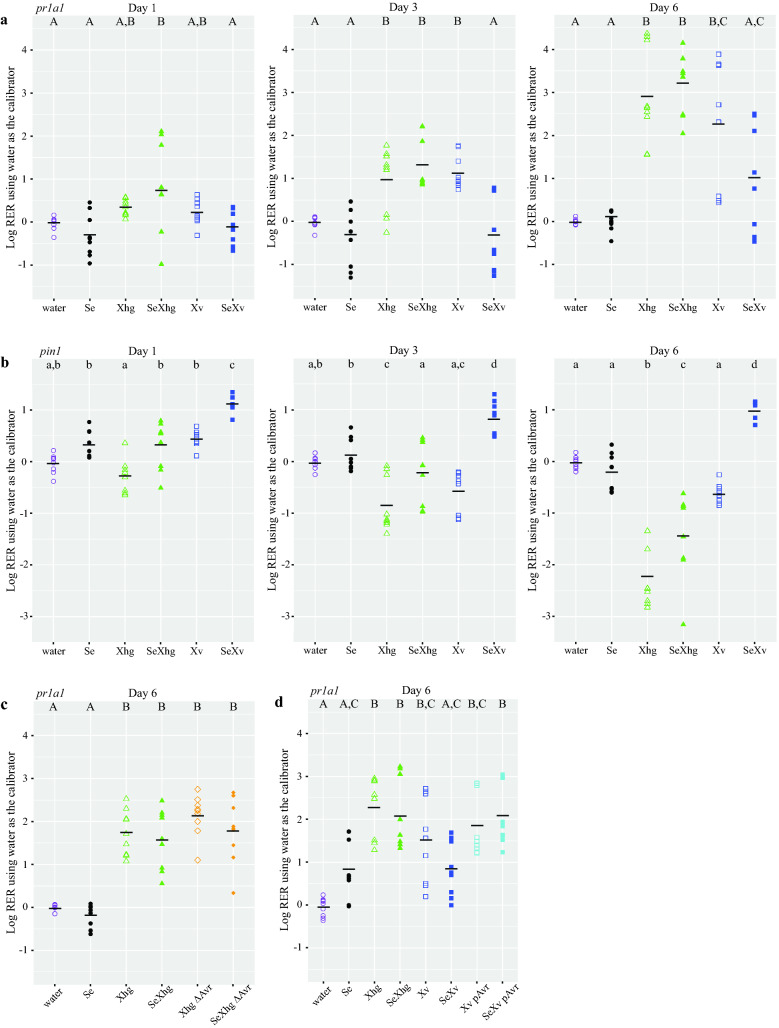
*Xanthomonas* species differentially impact the plant immune responses. Plant gene expression was quantified at days 1, 3, and 6 dpi with water (purple circles), *S. enterica* (Se; black circles), *Xhg* (Xhg; green, open triangles), *Xhg* + *S. enterica* (SeXhg; green, closed triangles), *X. vesicatoria* (Xv; blue, open squares), *X. vesicatoria* + *S. enterica* (SeXv; blue, closed squares), *Xhg avrHah1*^ΔDBD^ mutant (Xhg ΔAvr; open, orange diamonds), *S. enterica* + *Xhg avrHah1*^ΔDBD^ mutant (SeXhg ΔAvr; closed, orange diamonds), *X. vesicatoria* pUFR034 (*avrHah1*) (Xv pAvr; open, cyan squares), or *S. enterica* + *X. vesicatoria* pUFR034 (*avrHah1*) (SeXv pAvr; closed, cyan squares). Data for the SA-inducible *pr1a1* (**a**,**c**,**d**) and JA-inducible *pin1* (**b**) are displayed as log-transformed relative expression ratios (RER) using water-treated plants as the calibrator. Each symbol represents transcription levels in one tomato plant, and the dashed lines indicate a two-fold change relative to the water control. Means for each treatment at each time point are depicted with horizontal black lines. Transcription was measured in plants from three biological replicates, sampling from three plants per treatment per timepoint for each replicate. Letters denote significant differences between treatments within a single time point using Tukey’s HSD test (*P* < 0.05). Combining three independent experiments, n = 9 plants per treatment per time point.

At 1 dpi, there were few differences in *pr1a1* expression between treatments (Fig. 3a). The *Xhg* with *S. enterica* co-inoculation treatment was significantly different from the negative control (plants treated with water) (P = 0.0018), but all other treatments were statistically the same as the negative control at this time point (Fig. 3a). Compared to the negative control, tomatoes that were inoculated with *Xhg, Xhg* and *S. enterica*, or *X. vesicatoria* showed significant increases in *pr1a1* expression by 3 dpi (Fig. 3a; P = 0.0205, 0.0005, and 0.0032, respectively). Contrastingly, tomato plants treated with *X. vesicatoria* and *S. enterica* or *S. enterica* alone had no change in *pr1a1* expression compared to the water control at 3 dpi (Fig. 3a; P = 0.901 and 0.889, respectively). By 6 dpi, *pr1a1* levels had increased in plants inoculated with *Xhg, Xhg* and *S. enterica*, or *X. vesicatoria* with changes reaching approximately 100–10,000-fold compared to water controls (Fig. 3a; P = 5 × 10^−7^, < 1 × 10^−7^, and 7.46 × 10^−5^, respectively). Plants treated with *X. vesicatoria* and *S. enterica* or *S. enterica* alone had no change in *pr1a1* expression compared to the water control at 6 dpi (Fig. 3a; P = 0.244 and 1.00, respectively). Plants inoculated with *X. vesicatoria* and *S. enterica* had *pr1a1* levels that were statistically the same as both the water control and the *X. vesicatoria* alone treatment (Fig. 3a; P = 0.244 and 0.084, respectively).

At 1 dpi, *pin1* expression levels showed similar trends as *pr1a1.* There were few differences from the water control except for the *X. vesicatoria* with *S. enterica* treatment which showed tenfold higher levels of *pin1* expression (Fig. 3b; P < 1 × 10^−7^). By 3 dpi, treatment with *Xhg* led to reduced levels of *pin1* while treatment with *X. vesicatoria* and *S. enterica* resulted in increased *pin1* expression compared to the water control (Fig. 3b; P = 0.0011 and 0.00097, respectively). Although inoculation with *X. vesicatoria* or *S. enterica* alone had no change in *pin1* expression compared to the water-inoculated plants at 6 dpi (Fig. 3b; P = 0.071 and 0.939, respectively), inoculation with *Xhg* either with or without *S. enterica* led to a 1–3 log decrease in *pin1* levels (Fig. 3b; P < 1 × 10^−7^ and 3 × 10^−7^, respectively). Co-inoculation with *X. vesicatoria* and *S. enterica* led to a tenfold increase in *pin1* expression at 6 dpi (Fig. 3b, P = 0.00048). Taken together, these data show that the two *Xanthomonas* spp. induce different plant immune responses.

To determine if there was a connection between AvrHah1 and the observed induction of *pr1a1* transcription, we monitored *pr1a1* levels at 6 dpi in the *Xhg avrHah1*^ΔDBD^ mutant. Wildtype *Xhg* infection led to increased *pr1a1*expression (Fig. 3c; P = 1 × 10^−7^ and 5 × 10^−7^). Inoculation with the *Xhg avrHah1*^ΔDBD^ mutant also induced *pr1a1* expression compared to the water control (Fig. 3c; P < 1 × 10^−7^). Further, we measured both free and conjugated forms of SA in leaf tissue at 1 and 3 dpi after dip-inoculation with water, *S. enterica*, *Xhg, Xhg avrHah1*^ΔDBD^, *X. vesicatoria*, or *S. enterica* with each xanthomonad. SA levels between the treatments were not significantly different (P > 0.05; Fig. S1). These data demonstrate that *avrHah1* is not required for the induction of *pr1a1* during *Xhg* infection.

To examine the impact of AvrHah1 on plant gene expression in response to *X. vesicatoria,* leaf samples were taken at 6 dpi and examined for *pr1a1* expression. Compared to the negative control, tomatoes that were inoculated with *Xhg, Xhg* and *S. enterica*, *X. vesicatoria* + pURF034, *X. vesicatoria* + pUFR034 (*avrHah1*), *X. vesicatoria* + pUFR034 (*avrHah1*) and *S. enterica* showed significant increases in *pr1a1* expression by 6 dpi (Fig. 3d; P = 1 × 10^−7^, 8 × 10^−7^, 0.00048, 1.1 × 10^−5^, and 7 × 10^−7^, respectively). Contrastingly, tomato plants treated with *X. vesicatoria* + pURF034 and *S. enterica* or *S. enterica* alone had no change in *pr1a1* expression compared to the water control at 6 dpi (Fig. 3d; P = 0.165 and 0.174, respectively). Thus*,* addition of *avrHah1* to *X. vesicatoria* alters the immune response to resemble the host response to *Xhg*.

### AvrHah1 is not required for electrolyte leakage in *Xhg*-infected tomato leaves

Previous work has demonstrated that *Xhg* infection leads to more cellular damage in tomato leaves, as measured through electrolyte leakage, than *X. vesicatoria* infection^17^. From those data, it was hypothesized that this increase in cellular damage led to a resulting increase in *S. enterica* persistence in the phyllosphere^17^. Separately, it was shown that AvrHah1 is required for induction of electrolyte leakage in pepper leaves that have been infiltrated with *Xhg*^30^. To determine if AvrHah1 is linked to cellular damage and the resulting increases in *S. enterica* populations in tomato, plants were dip-inoculated with water, *S. enterica*, *Xhg, Xhg avrHah1*^ΔDBD^, *X. vesicatoria*, or *S. enterica* with each xanthomonad. Leaf samples were collected at 6 dpi. Plants treated with wildtype *Xhg* and *S. enterica* or *X. vesicatoria* with or without *S. enterica* had higher levels of electrolyte leakage than the water or *S. enterica* controls (P < 0.01, Fig. S2). The *Xhg avrHah1*^ΔDBD^ treatment resulted in intermediate electrolyte leakage levels that were statistically similar to the water and *S. enterica* controls and both the wildtype *Xhg* and *X. vesicatoria* treatments (P > 0.01; Fig. S2). The *Xhg avrHah1*^ΔDBD^ mutant and *S. enterica* treatment gave similar results as the *avrHah1*^ΔDBD^ mutant treatment except that it had statistically lower levels of electrolyte leakage than the two *X. vesicatoria* treatments (P < 0.01; Fig. S2). These results indicate that specific Xanthomonads cause different levels of electrolyte leakage, but these differences do not correlate with increased *S. enterica* persistence.

## Discussion

As foliar pathogens, *Xanthomonas* species survive on both the leaf surface and in the leaf interior. To succeed on the surface, epiphytic bacteria tolerate varying degrees of stress, including UV exposure, desiccation, and nutrient limitation. By actively moving to the leaf interior, xanthomonads can avoid these stresses and thrive. Two goals of this work were to identify which aspect(s) of the *Xhg* disease cycle benefit *S. enterica* and to characterize the mechanism that explains the observed phenotypic differences between *S. enterica* populations on *Xhg*- and *X. vesicatoria*-infected plants.

**Figure 4:**
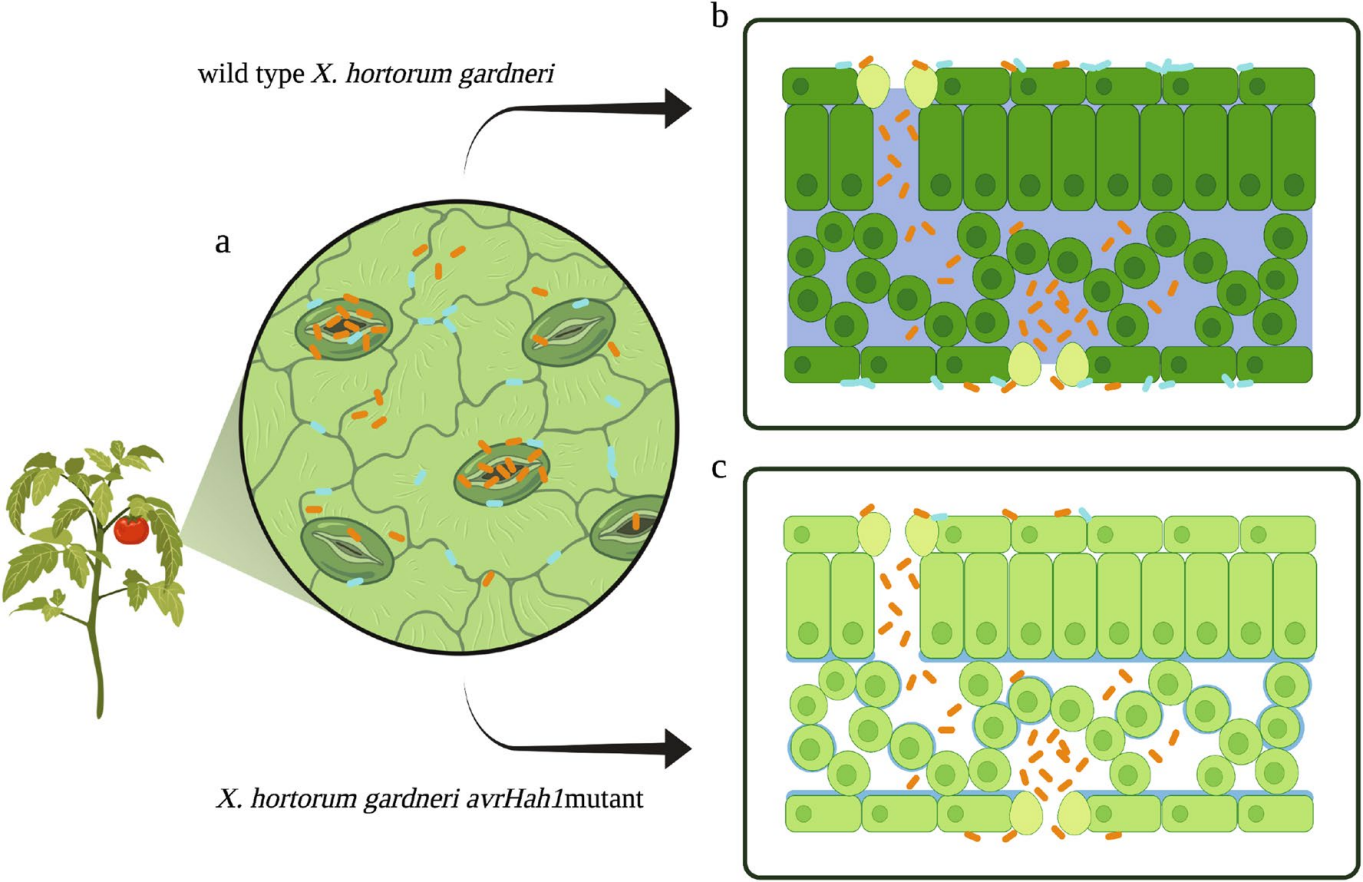
Model for *Xhg* enhancement of *S. enterica* persistence in water-soaked leaf tissue. Depiction of the tomato leaf surface (**a**) shows the location of bacterial cells (*Xanthomonas*, orange; *S. enterica*, cyan). *Xhg* tends to cluster at stomates (pores in the leaf surface) while *S. enterica* has a more random distribution with some cells at cell junctions (wavy lines), stomates (large ovals), and trichomes (not shown). Cross sections of the leaf (**b**,**c**) indicate entry of *Xanthomonas* bacterial cells (wildtype *Xhg,* **b**; *Xhg avrHah1*^ΔDBD^ mutant, **c**) into the apoplastic space through stomates and some of the resulting consequences to leaf physiology. Water soaking seen during infection by wildtype *Xhg* is depicted by the darker green plant cells and the increased amount of water and/or nutrients (purple) in the apoplast (**b**). This figure was adapted from “Leaf Surface Structure” and “Leaf Anatomy”, by BioRender (2021). Retrieved from https://app.biorender.com/biorender-templates.

For this study, we hypothesized that one or more modifications to the leaf caused by *Xhg* infection benefit other leaf community members and lead to increased *S. enterica* survival. The infection process includes aggregation on the leaf surface, entry into the leaf interior, promotion of a water-soaked apoplast, and suppression of host immunity (Fig. 4). Initial *S. enterica* attachment to the leaf surface appears to be independent of *Xhg* as there are no statistical differences between treatments for early *S. enterica* populations (Fig. 1a; 1 and 3 dpi). Primary lesions develop approximately three dpi and appear as small, circular lesions just visible on the underside of leaves and become more numerous with a water-soaked appearance at four dpi. Based on the rapid appearance of primary lesions, *Xhg* quickly modifies the leaf environment to create the macroscopically visible water-soaked lesions. The prevalence of the primary lesions and water-soaked areas continue to increase through six dpi, the final time point sampled in these experiments, but there is little to no growth in individual lesion size at that time. By six dpi, *Xhg*-treated plants have ten-fold more *S. enterica* than plants treated with *S. enterica* alone (Fig. 1a). The connection between the observed differences in *S. enterica* populations with the timing of disease progression suggests that the appearance or increased frequency of the water-soaked lesions resulted in increased *S. enterica* survival (Fig. 4). Alternatively, or concurrently, the SA-inducible *pr1a1* gene begins to show signs of induction as early as 1 dpi with *Xhg* (Fig. 3a). The rapid impact of *Xhg* on the plant immune response could manifest as increased *S. enterica* survival six days later. Regardless of the mechanism, it takes less than a week in the *Xhg* infection process to significantly affect the persistence of *S. enterica* in this environment.

As *Xhg* impacts *S. enterica* in the early stages of disease (Fig. 1), the T3SS effectors that have been linked to the acquisition of nutrients and the suppression or evasion of the plant immune response (for review, see^33^) could be factors that affect this process. We chose to focus on AvrHah1 because this effector has been linked to the water soaking phenotype in *Xhg* and is absent from the *X. vesicatoria* genome^26,30^. Thus, its specificity in *Xhg* made it a potential factor that could explain the different effects of these two species on *S. enterica* survival. As shown in this work, AvrHah1 is both necessary and sufficient for *Xanthomonas*-dependent increases in *S. enterica* persistence. The *Xhg avrHah1*^ΔDBD^ mutant is no longer beneficial towards *S. enterica* survival (Fig. 1), and *X. vesicatoria* carrying a plasmid-borne copy of *avrHah1* demonstrates a gain of function for this phenotype (Fig. 2). To characterize the mechanism by which AvrHah1 leads to increased *S. enterica* persistence, we examined downstream host targets of AvrHah1. Following dip-inoculation, we saw no significant changes in gene expression for *bHLH3, bHLH6, PL*, or *PE* (Table 1). This result contrasted with previous work demonstrating that infiltration of wildtype *Xhg* induced significantly higher transcription of these four genes compared to tomato plants that were infiltrated with the *Xhg avrHah1*^ΔDBD^ mutant^29^. The two studies differ in several aspects of experimental design, including *Xhg* strain, tomato genotype, and inoculation method (dip-inoculation vs infiltration). We found that infiltration of wildtype *Xhg* mildly induced expression of only one of the four genes, *bHLH6,* compared to the *Xhg avrHah1*^ΔDBD^ mutant (Table 1). An alignment of the predicted AvrHah1-binding sites upstream of *bHLH3* and *bHLH6* showed that the two tomato varieties (Heinz 1706 and MoneyMaker) are 100% identical in that region (data not shown). While these results do not completely preclude a role for tomato variety, they support the idea that bacterial strain likely impacts the observed differences in expression between experiments (Table 1; ^29^). Regardless, these data demonstrate that AvrHah1 plays a key role in the ability of *Xhg* to increase *S. enterica* persistence. In this system, AvrHah1 may have different targets that may influence the leaf environment. Schwartz et al. identified 4,106 potential AvrHah1 binding sites in tomato^29^. Future characterization of potential targets could provide a more detailed mechanism for AvrHah1-mediated increases in *S. enterica* persistence.

Pathogens, such as *Xhg*, have restricted access to the leaf interior through natural openings such as stomates, hydathodes, and wounds. Entry through stomates brings the bacteria to the apoplast, an air-filled, intercellular space^34,35^. During infection with wildtype *Xhg*, the apoplast is inundated with host cellular constituents, creating the water-soaked lesions seen in infected tomato plants (Fig. 4; ^30^). The water soaking phenotype is absent in plants infected with the *Xhg avrHah1*^ΔDBD^ mutant. A water-soaked apoplast leads to higher *Xanthomonas* populations, spread of pathogens into host tissues, suppression of host defense responses, and potentially promotes availability of nutrients^36^. As part of the defense response, plants cause local desiccation to limit pathogen replication (reviewed in^37^), and the AvrHah1-dependent water soaking may benefit *S. enterica* by reducing desiccation stress in this niche. In *Arabidopsis thaliana,* virulent *P. syringae* pv *tomato* DC3000 causes water potentials that promote pathogen growth while avirulent DC3000 are associated with higher levels of desiccation that inhibit bacterial growth^38,39^. In that study, desiccation was measured using a reporter fusion to the *proU* promoter, which was induced under low water potentials. Utilizing a reporter to monitor water potentials in future experiments could prove to be informative regarding the role of AvrHah1-mediated water soaking in resulting water stress levels.

In addition to desiccation, epiphytic bacteria also experience nutrient limitation on the leaf surface due to the cuticle that restricts diffusion of nutrients from inside the leaf^40^. Migration to the leaf interior may provide access to the many nutrients (sugars, amino acids, etc.) that are transported through the apoplast and into phloem tissue. Although carbon sources such as glucose, fructose, GABA, succinate, and others accumulate in the apoplast, they are typically sequestered in forms that are bound in the cell wall or within cellular vacuoles^41^. Thus, we hypothesized that *Xhg* water soaking could release cellular constituents, nutrients, and electrolytes in an accessible form due to changes in host cell membrane permeability (Fig. 4). This result would not be the first example where *S. enterica* utilizes host-derived molecules made available by other bacteria. Plant cell wall breakdown by the soft-rotting pathogen *Pectobacterium carotovorum* produces oligosaccharides that are thought to be scavenged by *S. enterica*^16,21^. There are also several examples of phytopathogens altering host cell membrane permeability for growth. *Pantoea stewartii* subsp. *stewartii* causes water-soaked lesions on corn leaves by damaging cell membranes to release water and nutrients^42^. Water soaking caused by the endophytic pathogen *P. syringae* pv. *phaseolicola* releases Ca^2+^, Fe^2/3+^, and Mg^2+^ into the apoplast of bean leaves^43^. Our conductance data suggests that electrolyte leakage does not correlate with increased *S. enterica* persistence. Plants co-inoculated with either *Xhg* or *X. vesicatoria* and *S. enterica* have increased electrolyte leakage compared to the water and *S. enterica* controls (Fig. S2). However, unlike *Xhg, X. vesicatoria* infection has no impact on *S. enterica* populations (Fig. 2). Thus, gross changes in electrolyte leakage cannot explain the *X-gardneri* specific effects on *S. enterica* survival. Although overall differences in electrolyte leakage do not appear to be correlated with *Xhg*-mediated increases in *S. enterica* survival, these experiments could have missed changes in the levels of specific electrolytes. Fluctuations in ions of low abundance, like iron, wouldn’t have been detected using this approach. Iron has repeatedly been identified as a host-limited nutrient, and pathogens, including *S. enterica*, have multiple mechanisms to scavenge iron from their environment^44–46^. An increase in iron availability could indicate that host manipulations by *Xhg* release this critical factor for *S. enterica* growth. Future experiments examining the release of specific ions could elucidate more details of this process.

Invading microorganisms face a number of plant defense responses (antimicrobials, reactive oxygen species (ROS), ‘pathogenesis related’ (PR) proteins, etc.) that must be overcome to survive in intercellular spaces. The differential ability of *S. enterica* to colonize a range of tomato cultivars suggests an active plant response to the enteric pathogen^7^. Leaf discs treated with the *S. enterica* flagellar epitope flg22 showed increased levels of ROS^47^, and dip-inoculation of tomatoes with *S. enterica* resulted in a transient induction of the SA-inducible *pr1a1* defense gene^14^. Other work has demonstrated that the presence of a virulent pathogen can protect another non-pathogenic epiphyte from host immunity. A nonpathogenic *X. euvesicatoria* T3SS mutant regains the ability to grow *in planta* if co-inoculated with pathogenic *X. euvesicatoria*^48^*.* The authors showed that the pathogenic *X. euvesicatoria* suppressed host immunity, prevented recognition of LPS from both bacterial strains, and inhibited host defense responses^48^. Further, manipulation of the immune system has been linked to water soaking and T3SS in other phytopathogens. *P. syringae* pv *tomato* induces water soaking and suppresses SA-dependent responses in *A. thaliana* using two T3SS effectors HopM1 and AvrE^49,50^. Recent work also demonstrated that HopM1 and AvrE induce abscisic acid (ABA) production to close stomata and produce water soaked lesions^51,52^. We hypothesized that *Xhg* may similarly manipulate the immune system, providing protection for *S. enterica* on tomato leaves. Co-inoculation of *S. enterica* with *Xhg* resulted in sustained induction of *pr1a1* and repression of the JA-inducible *pin1* (Fig. 3). As induction of *pr1a1* contrasts with suppression or no change in expression of *pr1* genes by other pathogens in wheat or *A. thaliana*^50,53^, respectively, our results suggest that *Xhg* host immune manipulation may occur through a different mechanism. Contrastingly to *Xhg*, co-inoculation of *S. enterica* with *X. vesicatoria* showed no changes in *pr1a1* transcription and induced expression of *pin1* (Fig. 3). Despite these differences in *pr1a1* gene expression between treatments, there was no effect on levels of free or conjugated SA at one and three dpi for any treatment (Fig. S1). Induction of the SA pathway typically results in a spike in hormone levels followed by a rapid return to basal levels^54^. Thus, it is possible that we missed a transient increase in SA that could have led to *pr1a1* induction in the *Xhg*-treated plants. Even a short burst of SA production could have beneficial effects on *S. enterica*. Although SA is normally considered to be a mediator of defense responses against bacteria, increased SA levels can result in increased *S. enterica* antibiotic resistance^55^ which could enhance *S. enterica* resistance to stresses in this niche. Alternatively, SA levels may remain constant, and *pr1a1* may be induced by a different signaling pathway, such as ethylene. However, because the *Xhg avrHah1*^ΔDBD^ mutant induced *pr1a1* expression to the same extent as wildtype *Xhg*, we concluded that *pr1a1* induction does not contribute to the role of AvrHah1 in increasing *S. enterica* persistence. Although AvrHah1 is not involved, the importance of SA and other plant hormones in *S. enterica* survival requires further study as there were still significant differences between *Xhg* and *X. vesicatoria* in these initial experiments.

In summary, this work identifies one *Xhg* factor, AvrHah1, which is both necessary and sufficient for providing conditions that lead to increased *S. enterica* persistence. Although the mechanism by which AvrHah1 promotes *S. enterica* survival remains unclear, several potential explanations have been described and will require further exploration. *Xhg* could provide increased nutrient availability through water soaking and/or defense against the plant immune response. Interestingly, *X. perforans* strains carrying the *avrHah1* gene have recently been identified in the southeastern United States, and these strains have gained the ability to cause water soaking in pepper leaves^56^. While *X. perforans* is typically found in the southeastern United States*, Xhg* is commonly isolated in the midwestern United States. The recent emergence of another species, with different geographic distribution, carrying *avrHah1* suggests selective pressure for horizontal transfer of the *avrHah1-*containing extrachromosomal plasmid between species. Acquisition of *avrHah1* may promote *Xanthomonas* disease and raises the concern that this genetic transfer could lead to an increased risk of *S. enterica* contamination of crops around the country. Continued research in this area will provide fundamental knowledge of how an inhospitable environment changes during infection, resulting in survival of human pathogens and an increased risk of human disease.

## Materials and methods

### Bacterial strains, media, and culture conditions

A kanamycin-resistant strain of *S. enterica* serovar Typhimurium 14028s and nalidixic acid strains of *X. vesicatoria* 1111 (ATCC 35937), and *Xhg* 444 (spontaneous Nal-resistant strain; this study) were used as wildtype strains in this study. *S. enterica* was made kanamycin resistant by inserting the *aadA* gene at the *att*Tn*7* site. The *Xhg avrHah1*^Δ*DBD*^ mutant was obtained from J. Jones^30^ and the *X. vesicatoria* pUFR034 and pUFR034 (*avrHah1*) strains were made by transforming *X. vesicatoria* 1111 with the Kanamycin-resistant plasmids pUFR034 or pURF034 (*avrHah1*) (^57^ and Minsavage, G., unpublished). Bacterial cultures were grown in lysogeny broth (LB) for *S. enterica* at 37 °C or nutrient broth (NB) for *Xanthomonas* spp. at 28 °C with shaking at 200 rpm. The antibiotics nalidixic acid (Nal) and Kanamycin (Kan) were used at concentrations of 20 and 50 µg ml^−1^, respectively. Strains used in this study are shown in Table 2.

**Table 2:** List of Strains and Primers.

Strain designation	Genotype	Reference or source	
JDB1022	*S. enterica* serovar Typhimurium 14028s; Kan^R^ at the *att*Tn*7* site	This study	
JDB1052	*X. vesicatoria* 1111	ATCC 35937	
JDB1470	*X. hortorum* pv. *gardneri* 444; Nal^R^	This study	
JDB1459	*X. hortorum* pv. *gardneri* 444 *avrHah1*^Δ*DBD*^ mutant (M28)	^30^	
JDB1481	*X. vesicatoria* 1111 + pUFR034; Kan^R^	This study	
JDB1482	*X. vesicatoria* 1111 + pUFR034 (*avrHah1*); Kan^R^	This study	

Primer	Primer sequence (5′ to 3′)		% Efficiency
	Forward	Reverse	
Quantitative PCR analysis			
*act41*	GCTCTTGACTATGAACAGGAAC	AAGGACCTCAGGACACCG	104
*ubi3*	GCCGACTACAACATCCAGAAGG	TGCAACACAGCGAGCTTAACC	105
*pin1*	GCTAAGGAAATAATTGAGAAGGA	TAAGTCACCACAGGCATT	102
*pr1a1*	TCAAAGAGCTGATGACTGTG	GTACCATTGCTTCTCATCGT	102
*bHLH3*	TGAGAAACAGGGTGATAATGGG	GTACCCTGTTGGTGCTTCTT	109
*bHLH6*	TGCAAGAGCTTTCTGTCAATATG	TAGAGCAGAGGGAGGAAGAA	102
*PL*	TGATTGTGCAATTGGCTTTGG	TGTTCCTGGCTTTGGATTTACT	103
*PE*	ACAATCTCCCAAGCACAAGG	GCATATGGGAGAAGGGTGTTTAG	97

### Plant inoculation

Tomato cultivar MoneyMaker seeds were purchased commercially (Eden Brothers). No plant material was collected. This study complies with the relevant institutional, national, and international guidelines and legislation for experimental research on plants. Seedlings were cultivated in Professional Growing Mix (Sunshine Redi-earth) with a 16 h photoperiod at 24 °C for five weeks. For colonization assays, *Xanthomonas* bacterial cultures were grown for two days in NB (or NB with Kan for pUFR strains) at 28 °C, and *S. enterica* cultures were grown overnight in LB at 37 °C. Bacterial strains were normalized to an optical density at 600 nm (OD_600_) of 0.2 (for *S. enterica* and *X. vesicatoria* strains), 0.25 (for the *Xhg avrHah1*^Δ*DBD*^ mutant), and 0.3 (for wildtype *Xhg*) in sterile water. These OD_600_ values correspond to a bacterial population level of ~ 10^8^ CFU/ml for the respective strains. Normalized cultures were diluted 1:100 in sterile water for an inoculum level of ~ 10^6^ CFU/ml. Treatments consisted of individual bacterial strains mixed with equal parts water (*S. enterica*, *Xhg*, *X. vesicatoria*, *Xhg avrHah1*^Δ*DBD*^ mutant, *X. vesicatoria* pUFR034, or *X. vesicatoria* pUFR034 (*avrHah1*)), *S. enterica* mixed 1:1 with each individual *Xanthomonas* strain, or water alone. Prior to inoculation, 0.025% Sil-Wett was added to water or the bacterial inoculum. Tomato plants were dip-inoculated by inverting plants in either sterile water or the bacterial inoculum for 30 s with agitation to prevent bacterial cell settlement. Plants were incubated at high humidity for 48 h in lidded, plastic bins under grow lights with a 16 h photoperiod at room temperature (~ 26 °C). After 48 h, plants were exposed to low humidity conditions (bin lids were removed) during the day and high humidity conditions (bin lids were replaced) during the night. At multiple time points post-inoculation, leaf samples were taken using destructive sampling to determine bacterial populations or collect samples for tomato RNA extraction. For infiltration experiments, MoneyMaker tomato leaves were infiltrated with *Xhg* wildtype or the *avrHah1*^ΔDBD^ mutant at OD_600_ = 0.25, following published protocols^29^. At 48 h post-infiltration, two 79 cm^2^ leaf discs were taken from each of two leaflets on middle leaves and combined for a total of four leaf discs per plant. Samples from four plants per treatment were collected and frozen at −80 °C for further processing, as described below.

### Bacterial population sampling

Bacterial populations on leaves were determined as described^14^. Briefly, at indicated times, one 79 cm^2^ leaf disc was taken from a leaflet on middle leaves using a surface sterilized cork borer^18^. Samples from three plants per treatment per time point were individually homogenized in 500 µl of sterile water in microfuge tubes using a 4.8 V rotary tool (Dremel, Mt. Prospect, IL) with microcentrifuge tube sample pestle attachment (Fisher Scientific). Homogenates were diluted as needed and spiral plated (Autoplate 4000, Spiral Biotech, Norwood, MA) on LB Kan (for *S. enterica*) or NB Nal or NB Kan Nal (for *Xanthomonas* spp.) plates. Resulting colonies were counted after overnight incubation at 37 °C (for *S. enterica*) or after incubation for three days at 28 °C (for *Xanthomonas* spp.) to determine bacterial populations. Although not shown in the figures, bacterial samples were taken at Day 0 to confirm that all bacterial populations were equivalent at the beginning of each experiment. No statistical differences were noted between treatments for any experimental setup at Day 0 (P > 0.01). Experiments were performed with three biological replicates.

### RNA isolation

Tomato gene expression levels were determined as described^14^. At indicated time points, two 79 cm^2^ leaf discs were taken from each of two leaflets on middle leaves and combined for a total of four leaf discs per plant. Samples from three plants per treatment per time point were collected and frozen at −80 °C for further processing. RNA was extracted using the Maxwell® RSC Plant RNA kit (Promega). Briefly, four leaf discs were homogenized with a mortar and pestle in the presence of liquid nitrogen. Ground tissue was transferred to a small weigh boat containing 450 µl homogenization solution, mixed with a pipette tip, and transferred to a microcentrifuge tube. Samples were then either stored at −80 °C until further processed or immediately processed according to manufacturer’s instructions using a Maxwell® RSC (Promega). RNA samples were eluted in 50 µl volume of RNase-free water and were quantified by NanoDrop (Thermo Scientific). RNA was isolated from three technical replicates for each of three biological replicates.

### cDNA synthesis and real-time PCR

cDNA synthesis and real-time PCR were performed as described^14,58,59^. Briefly, cDNA was synthesized using the iScript cDNA synthesis kit (Bio-Rad). Real-time PCR primers for *pin1, pr1a1, bHLH3, bHLH6, PL,* and *PE* were designed with Beacon Designer software (Premier Biosoft International) avoiding template secondary structure (Table 2). Primer efficiencies (Table 2) were calculated using serial dilutions of MoneyMaker genomic DNA and CFX Manager 3.0 software (Bio-Rad). Reference transcripts and primers were chosen based on published works: *act41* and *ubi3*^60^. Stable expression between treatments was validated using the Best Keeper program and three independent RNA samples from each treatment per biological replicate. Real-time PCR experiments utilized the CFX96 Real-Time System, and data were analyzed with the CFX Manager 3.0 software (Bio-Rad). The mean *Cq* of each target transcript was normalized by the mean *Cq* of each reference gene using the formula: 2^(−(*Cq* target-*Cq* reference))^. As previously described^60^, we determined the relative expression ratio (RER) of the target gene by dividing the normalized target RNA by a calibrator consisting of the average of the normalized values of the control samples (expression after water treatment in most experiments; expression after infection with wildtype *Xhg* in the AvrHah1 target experiments (Table 1)).

### Measurement of free and conjugated SA

Plants were inoculated with *S. enterica*, *Xhg*, *X. vesicatoria*, *Xhg avrHah1*^Δ*DBD*^ mutant, with *S. enterica* mixed 1:1 with each individual *Xanthomonas* strain, or with water alone, as described above. Leaf tissue samples were collected at multiple time points post-inoculation, weighed, flash frozen in liquid nitrogen, and extracted in 600 µl of H_2_O:1-propanol:HCL (1:2:0.005) with 200 ng of d4-SA as an internal standard. For total SA, 2 µl of glacial HCL was added, and the sample boiled for 30 min as in ^53^. Both free and total SA were phase partitioned with 1 ml MeCl_2_ and the MeCl_2_:1-propanol layer was collected and derivatized with trimethylsilydiazomethane, collected by vapor phase extraction on SuperQ columns, eluted in MeCl_2_and analyzed using GC–MS as in^61^.

### Electrolyte leakage

Plants were inoculated with *S. enterica*, *Xhg*, *X. vesicatoria*, *Xhg avrHah1*^Δ*DBD*^ mutant, with *S. enterica* mixed 1:1 with each individual *Xanthomonas* strain, or with water alone, as described above. Leaf tissue samples were collected at 6 dpi for bacterial populations (as described above) and electrolyte leakage measurements. Three leaf discs taken from a leaflet from a middle leaf were pooled from each plant, and changes in electrolyte leakage (conductance) were quantified as previously described^15^.

### Statistical analysis

All statistical analyses were performed using R software (version 2.14.1; R Development Core Team, R Foundation for Statistical Computing, Vienna, Austria [http://www.R-project.org]) as described^62^. Briefly, three biological replicates were performed for each experiment, and samples taken from one replicate were considered as subsamples. Linear regression analysis was used to determine whether bacterial population results differed between treatments for *S. enterica* and *Xanthomonas* spp. For real-time PCR analysis, three samples were compared for each treatment at each time point using Tukey’s HSD test. Results were considered statistically significant at *P* < 0.01.

## Supplementary Information

Supplementary Figures.

## Data Availability

All data generated or analyzed during this study are included in this published article (and its Supplementary Information files).
