# Supplementary material for: Xanthomonas hortorum pv. gardneri TAL effector AvrHah1 is necessary and sufficient for increased persistence of Salmonella enterica on tomato leaves
Source: Sci Rep. 2022 May 4;12:7313. doi: 10.1038/s41598-022-11456-6 (PMC9068798; doi:10.1038/s41598-022-11456-6)
Supplement: Supplementary file 1 — Supplementary Figures. [file 41598_2022_11456_MOESM1_ESM.pdf]

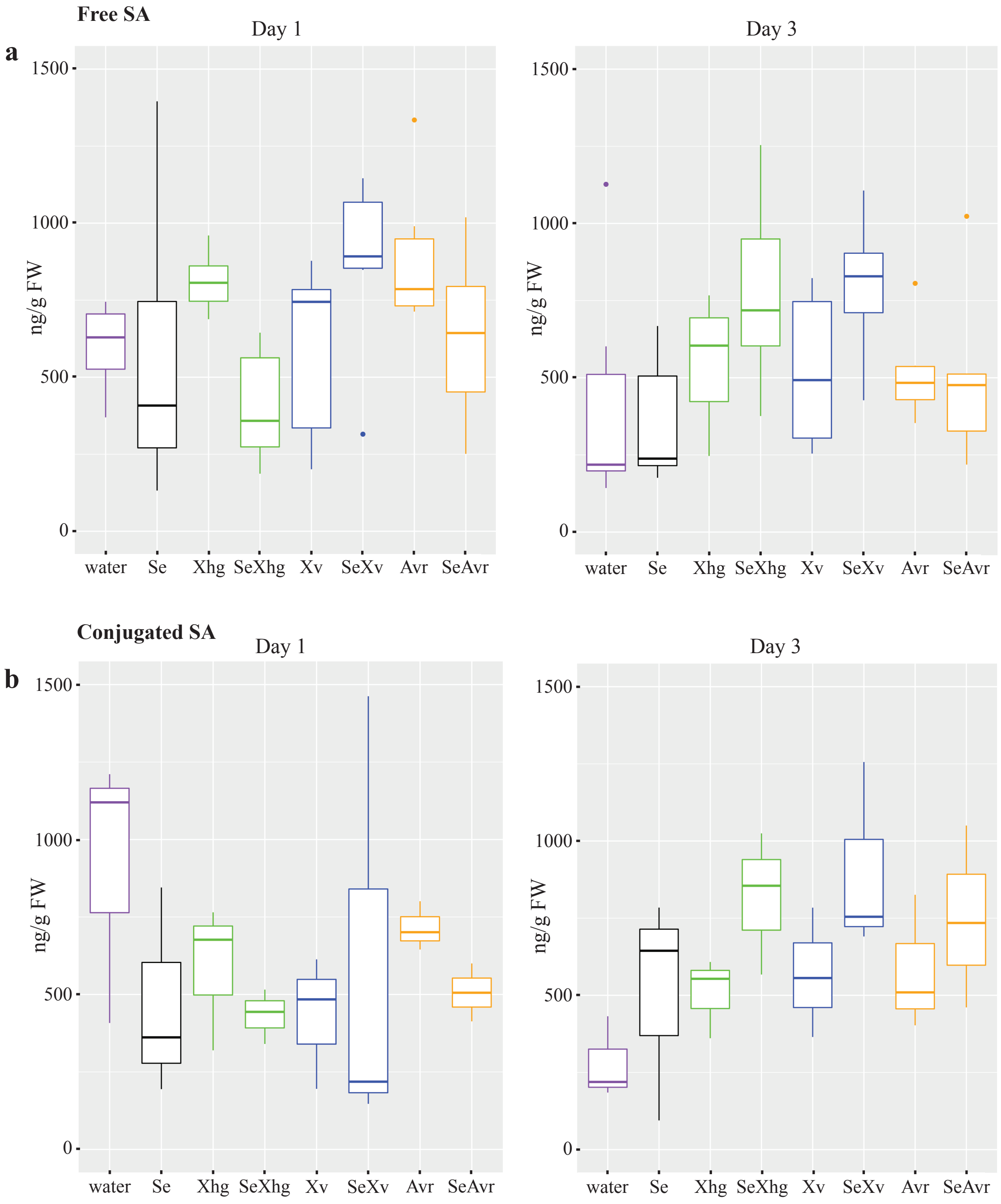

Figure S1. SA levels do not vary at Days 1 and 3 post-inoculation. Free (**a**) and conjugated (**b**) SA was quantified at Days 1 and 3 dpi with water (purple), *S. enterica* (Se; black), *Xhg* (Xhg; green), *S. enterica* + *Xhg* (SeXhg; green), *X. vesicatoria* (Xv; blue), *S. enterica* + *X. vesicatoria* (SeXv; blue), *Xhg avrHah1*$^{\Delta DBD}$ mutant (Avr; orange), or *S. enterica* + *Xhg avrHah1*$^{\Delta DBD}$ mutant (SeAvr; orange). SA levels are displayed as ng per gram of fresh weight tissue. Free and conjugated forms of SA were measured in plants from two biological replicates, sampling from three plants per treatment per timepoint for each replicate. There are no significant differences between treatments (P>0.05). Combining two independent experiments, n=6 plants per treatment per time point.

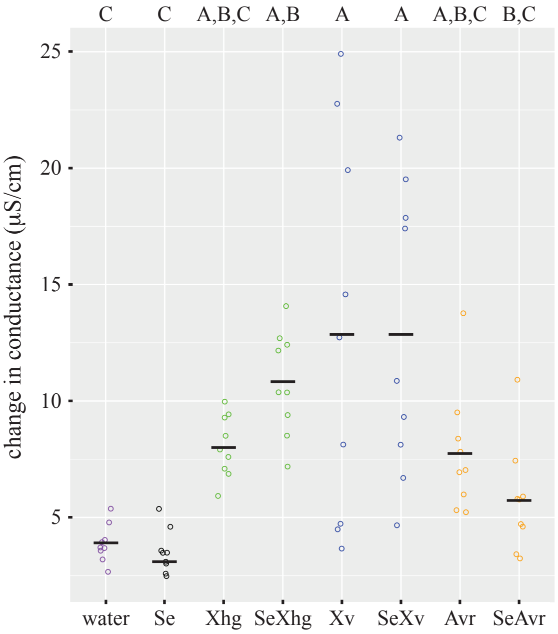

Figure S2. Electrolyte leakage does not correlate with treatments that benefit *S. enterica*. Electrolyte leakage (change in conductance) was quantified at 6 dpi with water (purple circles), *S. enterica* (Se; black circles), *Xhg* (Xhg; green circles), *S. enterica* + *Xhg* (SeXhg; green circles), *X. vesicatoria* (Xv; blue circles), *S. enterica* + *X. vesicatoria* (SeXv; blue circles), *Xhg avrHah1*$^{\Delta DBD}$ mutant (Avr; orange circles), or *S. enterica* + *Xhg avrHah1*$^{\Delta DBD}$ mutant (SeAvr; orange circles). Each circle represents electrolyte leakage levels in one tomato plant. Electrolyte leakage was measured in plants from two biological replicates, sampling from 3 or 6 plants per treatment per timepoint in the first and second replicate, respectively. Means for each treatment at each time point are depicted with horizontal black lines. Letters denote significant differences between treatments using Tukey's HSD test (P<0.01). Combining the two independent experiments, n=9 plants per treatment per time point.
